# Somatic growth in the first six months of life of infants exposed to maternal smoking in pregnancy

**DOI:** 10.1186/s12887-017-0819-7

**Published:** 2017-03-09

**Authors:** Mariana Lopes de Brito, Marina Nunes, Juliana Rombaldi Bernardi, Vera Lúcia Bosa, Marcelo Zubaran Goldani, Clécio Homrich da Silva

**Affiliations:** 1 Graduate Program in Child and Adolescent Health, Universidade Federal do Rio Grande do Sul (UFRGS), Porto Alegre, Brazil; 2 Departament of Nutrition, Universidade Federal do Rio Grande do Sul (UFRGS), Porto Alegre, Brazil; 3 Department of Pediatrics, Hospital de Clínicas de Porto Alegre, Porto Alegre, Brazil; 4 Center for the Study of Child and Adolescent Health, Porto Alegre, Brazil

**Keywords:** Pregnancy, Smoking, Intrauterine growth restriction, Infants, Growth, Longitudinal studies

## Abstract

**Background:**

Some studies suggest a relationship between maternal smoking during pregnancy and not only intrauterine fetal growth restriction or low birth weight, but also with changes in the postnatal growth and development. The objective of the present study was to investigate the effects of smoking during pregnancy on infants growth in the first 6 months of life compared with a control group and a group with idiopathic intrauterine growth restriction.

**Methods:**

Longitudinal observational study using a convenience sample of newborns divided into three groups: infants of smoking mothers (tobacco), with idiopathic intrauterine growth restriction (IUGR) and a control group. The sample was selected from two hospitals in Porto Alegre, located in southern Brazil, between 2011 and 2015. Newborns were evaluated at birth, 7 and 15 days, and in the first, third, and sixth month. Anthropometric measures were weight, length and head circumference. The growth indicators used were expressed as z-scores. The analyses were performed using the generalized estimating equation method.

**Results:**

The sample included 273 mother/newborn pairs: 86 tobacco group, 34 IUGR group, and 153 control group. In terms of weight at birth, all groups differed significantly (*p* < 0.001). The birth length of tobacco and control groups were similar, but the IUGR group was lower than both (*p* < 0.001). We found no differences in growth trajectory between tobacco and control group, but there were differences in the growth of the IUGR group when compared with the other groups. At 6 months of age, all groups had similar anthropometric measurements.

**Conclusion:**

Intrauterine growth restriction had major impact on the growth trajectory of the infants studied, regardless of other factors, such as smoking and diet.

## Background

Because of its high prevalence obesity has become a worldwide epidemic in recent years. The association of maternal smoking with the development of childhood obesity has been demonstrated in several studies. Two meta-analyses of observational studies in population of 3–33 months reported odds ratios of approximately 1.5 for overweight in the children of smoking mothers [1, 2]. In general, about 40% of the children in the world are exposed to smoking in the home. Environmental exposures occurring within a critical window of development, such as pregnancy or lactation, may trigger permanent changes to the metabolism, leading to diseases in adulthood, a phenomenon currently called programming [3].

It is estimated that 250 million women smoke on a daily basis worldwide. Despite the well-known harm many women do not quit smoking during pregnancy. In pregnant women, the nicotine and carbon monoxide components of cigarette smoke may cause damage to both the mother and the fetus, crossing the placental barrier [4].

Smoking during pregnancy is one of the greatest public health problems in the world due to its high prevalence and deleterious consequences. Smoking leads the preventable causes of unfavorable pregnancy outcomes and increased risk of morbidity and mortality in newborns [5, 6]. In Brazil, the prevalence of smoking during pregnancy ranges from 23 to 25%. The main maternal risk factors are Caucasian race, low socioeconomic status, low educational level, and single marital status [7].

Smoking has been associated with shorter duration of pregnancy miscarriages, and shorter duration of breastfeeding. During pregnancy, smoking is also a risk factor for intrauterine growth restriction (IUGR), perinatal mortality, low birth weight (LBW), and neurological abnormalities [8].

Several studies have shown an association between smoking during pregnancy and the development of obesity and hypertension in childhood and adolescence. However there is less consistent evidence concerning the association of maternal smoking during pregnancy and postnatal growth. Some authors have demonstrated the potential effect of tobacco smoke exposure and the development of childhood obesity [9–11]. More recently, other authors have shown the presence of stunting in children exposed to tobacco smoke during the intrauterine period [12–14].

The objective of the present study was to investigate the effects of smoking during pregnancy on infants’ growth in the first 6 months of life compared with a control group and a group with idiopathic intrauterine growth restriction.

## Methods

This is a longitudinal observational study using a convenience sample of mothers and their newborns divided into groups according to maternal exposures occurring during pregnancy. These groups were observed during the first semester of their children’s lives.

### Participants

The sample was selected from the Hospital de Clínicas de Porto Alegre and Hospital Femina of the Grupo Hospitalar Conceição located in the city of Porto Alegre, capital city of the state of Rio Grande do Sul (Brazil), from September 2011 to August 2015. The information about gestational age was collected from medical hospital records through of last menstrual period and precocious ultrasonography. There were included only pregnancy to term in this study. The mother/newborn pairs were divided into three groups: 1) smoking mothers (tobacco group): mothers who reported they smoked during pregnancy, regardless of the duration of exposure or the number of cigarettes smoked; 2) newborns who had idiopathic intrauterine growth restriction (IUGR group): newborns who had a birth weight below the 5th percentile according to the fetal growth curve proposed by Alexander et al. [15] and 3) control group: included nonsmoking mothers without diseases and those whose newborns did not have IUGR. These pairs did not have any diseases, liked gestational diabetes and hypertension, lived in the city of Porto Alegre, and the infants were delivered at the hospitals participating in the study. We excluded mothers who tested positive for HIV (Human Immunodeficiency Virus), twin infants, and newborns who require hospitalization and/or with acute diseases and/or congenital birth defects.

The use of an IUGR group may be considered as a second control group because generally children exposed to maternal smoking have lower birth weight and length. The combination of weight to be recovered and persistent height deficits could result in higher body mass index in children of smoking mothers [16].

### Data collection

The mother/newborn pairs were evaluated at birth 7 and 15 days, and in the first, third, and sixth month of life. A detailed description of the methods used in the present study has been published previously [17]. The anthropometric measures taken in all medical visits were weight, length, and head circumference. The following indicators were expressed as z-scores according to the WHO (World Health Organization) growth curves [18]: weight-for-age, weight-for-length, length/height-for-age and head circumference-for-age.

### Diet

The type of food given to the child was recorded using a 24-h diet recall and a questionnaire about the introduction of food. The instruments were administered in all interviews.

### Anthropometric data

Two investigators previously trained were taken the anthropometric measures. They used standardized techniques and calibrated equipment with the purpose of reducing interobserver and intraobserver variability. Infants’ body weight was measured in kilograms using a portable digital electronic scale (Marte® Scientific, São Paulo, Brazil) that was accurate to within 50 g. Each infant’s weight was then calculated by subtracting the mother’s weight from the total weight of mother and child combined. Infants’ length was measured in supine position, on a flat and stable surface, such as a table, using a portable stadiometer (Alturexata®, Belo Horizonte, Brazil). Children’s head circumference was measured around the largest occipital-frontal diameter across the forehead, just above the ears, using a non-stretch tape measure (Lange®, Ann Arbor, United States).

### Statistical analysis

The primary outcome we analyzed consisted of growth in the first 6 months of life. Our results were described and expressed as mean and standard deviation to evaluate the distribution of variables. The Kolmogorov-Smirnov test was used to assess normality. The chi-square test was used to assess the associations between categorical variables; whereas the *t*-test was used for parametric continuous variables. We used the generalized estimated equation (GEE) method which is based on the generalized linear model, to assess the growth trajectory of each group during the follow-up period and after, the Bonferroni multiple comparison test was used.

The significance level was set at 5% and the statistical analyses were performed using the Statistical Package for the Social Sciences (SPSS), version 18.0.

### Ethical aspects

The project was submitted to the Research Ethics Committee at the Hospital de Clínicas de Porto Alegre and Grupo Hospitalar Conceição. The project was approved under the protocol numbers 110097 and 11027 respectively.

## Results

Data collection was conducted between September 2011 and August 2015. Our sample included 273 mother/newborn pairs. Of these 34 pairs were allocated to the IUGR group, 86 pairs were included in the tobacco group, and 153 pairs formed the control group.

The distribution of sociodemographic perinatal, and anthropometric variables between groups is shown in Table 1. The education level of tobacco group was significantly lower than the other groups (*p* = 0.036). In terms of weight at birth, all groups differed significantly (*p* < 0.001). There was no difference between the tobacco group and the control group, but the weight of the IUGR group was lower than the groups (*p* < 0.001), regarding length and head circumference.

**Table 1:** Maternal sociodemographic, perinatal, and anthropometric characteristics according to group. IVAPSA cohort, Porto Alegre, September 2011–August 2015

	IUGR (34^**^)		TOBACCO (86^**^)		CONTROL (153^**^)		TOTAL (273)	
*Sociodemographic*								
Ethnicity, n (%)								
White	16	(47.1)	50	(58.1)	92	(60.1)	158	(57.9)
Black	12	(35.3)	22	(25.6)	32	(20.9)	66	(24.2)
Others^a^	6	(17.6)	14	(16.3)	29	(19.0)	49	(18.0)
Marital status, n (%)								
Living with a partner	29	(85.3)	58	(67.4)	129	(84.3)	216	(79.1)
Single	5	(14.7)	28	(32.6)	24	(15.7)	57	(20.9)
Educational level, n (%)								
≤ 8 years	9	(26.5)	43	(51.2)	49	(32.9)	101	(37.8)
9 < 11 years	22	(64.7)	38	(45.2)	89	(59.7)	149	(55.8)
≥ 12 years	3	(8.80)	3	(3.60)	11	(7.40)	17	(6.40)
Social class, n (%)								
Hight	10	(31.3)	21	(25.9)^b^	60	(41.4)	59	(31.7)
Middle	17	(53.1)	52	(64.2)	73	(50.3)	106	(57.0)
Low	5	(15.6)	8	(9.9)	12	(8.30)	21	(11.3)
Age, x ± SD	23.79	(7.07)	24.29	(6.24)	25.79	(6.79)	25.06	(6.79)
*Perinatal*								
Mode of delivery, n (%)								
Cesarean section	12	(35.3)	21	(24.4)	45	(29.6)	78	(28.7)
Vaginal delivery	22	(64.7)	65	(75.6)	107	(70.4)	194	(71.3)
Child gender, n (%)								
Female	20	(58.8)	42	(48.8)	86	(56.2)	148	(54.2)
Male	14	(41.2)	44	(51.2)	67	(43.8)	125	(45.8)
Mean Apgar–5 min, x (±SD)	9.64	(0.48)	9.52	(0.62)	9.46	(0.59)	9.50	(0.59)
Mean weight–grams (±SD)	2521.35	(29.42)	3071.45	(57.02)	3385.59	(36.78)	3179.39^*^	(32.50)
Mean length–cm (±SD)	46.121	(0.28)	48.012	(0.24)	49.306	(0.16)	48.511^*^	(0.14)
Mean head circumference–cm (±SD)	31.922	(0.21)	33.717	(0.14)	34.060	(0.12)	33.695^*^	(0.09)
*Anthropometric*								
Pre-pregnancy BMI, n (%)								
Underweight (<18.5 Kg/m^2^)	0	(0.00)	1	(2.10)	2	(1.90)	3	(1.70)
Normal weight (≥8.5 ≤ 24.9 Kg/m^2^)	19	(76.0)	25	(52.1)	62	(58.5)	106	(59.2)
Overweight (>24.9 ≤ 29.9 Kg/m^2^)	2	(8.00)	13	(27.1)	29	(27.4)	44	(24.6)
Obese (> 29.9 Kg/m^2^)	4	(16.0)	9	(18.8)	13	(12.3)	26	(14.5)
Gestational weight gain (Kg) (SD)	11.53	(5.80)	14.00	(6.89)	13.41	(6.71)	13.36	(6.68)

^a^Others: asian, brown and native brasilian

^b^The education level of tobacco group was significantly lower than the other groups (*p* = 0.036)

^*^ The perinatal variables birth weight, birth length, and head circumference at birth were statistically significant (*p* < 0.001). The other variables **did not** show statistically difference between the groups (*p* > 0.05 in the chi-square test for categorical variables and Student’s *t* test for parametric continuous variables)

^**^ Number (proportion) of missing data: Educational level *n* = 6 (2,19%), social class *n* = 15 (5.5%), mode of delivery *n* = 1 (0.36%), Mean apgar 5 min *n* = 3 (1,09), Mean weight *n* = 1 (0,36), Mean length *n* = 4 (1,46), Mean head circumference *n* = 7 (2,56), pre-pregnancy BMI *n* = 94 (34,43%), and gestational weight gain *n* = 38 (13.91%)

The average consumption and the median of cigarettes a day was respectively 10 and 11 cigarettes in the tobacco group. These similar values show that the dose consumed was similar among individuals in the group. Additionally, a national survey found that the average number of cigarettes consumed by the Brazilian is 12.9 cigarettes per day [19]. The sociodemographic characteristics also make us believe that consumption was similar, a time statistically significant differences were no found.

**Fig. 1:**
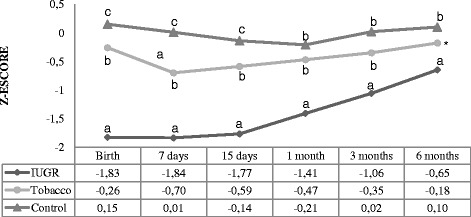
Weight-for-age z-score. Analysis performed using the generalized estimated equation (GEE) model. - Comparison between the groups (*P* < 0.001), comparison between times (*P* < 0.001), and comparison between group and times (interaction: *P* < 0.001). - We used letters a, b, and c to express the differences between the three groups. Means followed by the same letter showed no statistically significant difference (*P* > 0.05) based on the GEE analysis, followed by the Bonferroni multiple comparison test. * There was no statistically significant difference between the tobacco group and the other groups

The weight-for-age indicator is shown in Fig. 1 where it was observed a statistically significant difference between the three groups–intergroups (*p* < 0.001)–and when the measures were taken–intragroups (*p* < 0.001). At birth, all groups differed significantly in mean z-score. This difference persisted until the fifteenth day. At 1 and 3 month only the IUGR group differed. In the sixth month, the IUGR group only differed the control group (*p* = 0.03).

**Fig. 2:**
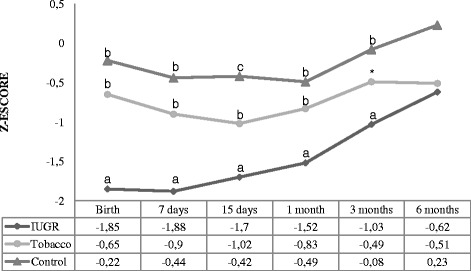
Length/height-for-age z-score. Analysis performed using the generalized estimated equation (GEE) model. - Comparison between the groups (*P* < 0.001), comparison between times (*P* < 0.001), and comparison between group and times (interaction: *P* < 0.001). - We used letters a, b, and c to express the differences between the three groups. Means followed by the same letter showed no statistically significant difference (*P* > 0.05) based on the GEE analysis, followed by the Bonferroni multiple comparison test. * There was no statistically significant difference between the tobacco group and the other groups

The length/height-for-age indicator is shown in Fig. 2 where we demonstrated a statistically significant difference intergroups (*p* <0.001) and intragroups (*p* < 0.001). At birth, the infants in the IUGR group differed from those other groups (*p* < 0.001), showing lower mean z-score. At 7 and 15 days the IUGR and tobacco group differed from control group. At 1 month, all groups differed statistically significantly. At 3 months, the IUGR and control group showed differences (*p* = 0.00). There was no difference between the groups at 6 months of age.

**Fig. 3:**
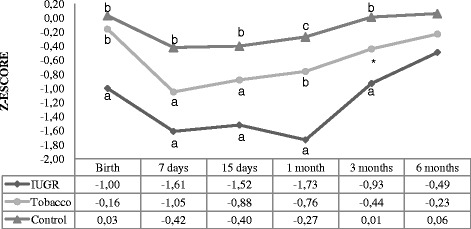
Weight-for-length z-score. Analysis performed using the generalized estimated equation (GEE) model. - Comparison between the groups (*P* < 0.001), comparison between times (*P* < 0.001), and comparison between group and times (interaction: *P* < 0.001). - We used letters a, b, and c to express the differences between the three groups. Means followed by the same letter showed no statistically significant difference (*P* > 0.05) based on the GEE analysis, followed by the Bonferroni multiple comparison test

The weight-for-length indicator is shown in Fig. 3 where the IUGR group differed from the others at birth. At 7 days all groups differed statistically significantly. At 15 days the IUGR and tobacco group differed from control group. After that, there was no difference of the measures between the groups.

**Fig. 4:**
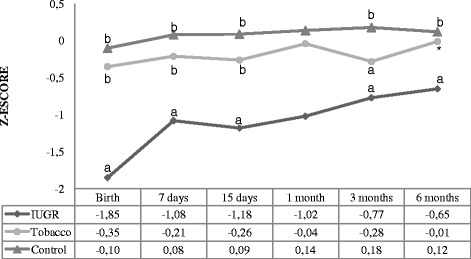
Head circumference-for-age z-score. Analysis performed using the generalized estimated equation (GEE) model. - Comparison between the groups (*P* < 0.001), comparison between times (*P* < 0.001), and comparison between group and times (interaction: *P* < 0.001). - We used letters a, b, and c to express the differences between the three groups. Means followed by the same letter showed no statistically significant difference (*P* > 0.05) based on the GEE analysis, followed by the Bonferroni multiple comparison test. *There was no statistically significant difference between the tobacco group and the other groups

The head circumference-for-age indicator is shown in Fig. 4 where the newborns of the IUGR group differed from those of the tobacco group (*p* < 0.001) and the control group (*p* < 0.001). At 7 days, all groups differed statistically significantly. At 15 days the IUGR group and the tobacco group showed similar measures; however these groups differed from the control group. At 1, 3 and 6 months, there was no difference between the groups.

In terms of diet during the first 6 months of life we found that the frequency of exclusive breastfeeding decreased during the follow-up period similarly in the three groups. Conversely, the artificial feeding increased with age. The introduction of solid or semisolid foods occurred similarly in the tobacco group and the control group after 15 days of life. This variables did not show statistically significant differences between the groups (*p* > 0.05).

## Discussion

The present study showed that the growth trajectory of children exposed to maternal smoking during pregnancy was similar to the control group. However as well as other studies, newborns exposed to tobacco showed differences in the weight-for-age at birth when compared with the control group, presenting a mean reduction of 314 g in birth weight.

In a study conducted by Suzuki et al. [20] the authors found that maternal smoking was a significant predictor of LBW in both genders, thus increasing the risk by 3.2 times. Similarly, Vardavas et al. [21] reported that smoking during pregnancy was associated with a reduction between 120 and 150 g in birth weight.

Epidemiological studies have shown that adverse environmental factors during pregnancy may cause IUGR and LBW thus leading to the potential development of metabolic syndrome. Anthropometric measures, such as birth weight, are influenced by genetic and epigenetic factors combined with the intrauterine environment [22]. It is possible to assume that the relationship between smoking during pregnancy and overweight in childhood may be mainly related to IUGR and LBW. Such hypothesis is based on the fact that, in the present study, it was observed that newborns in tobacco group did not show LBW and their growth trajectory was similar to controls despite their exposure to tobacco smoke.

Despite others studies in children and adults had showed the association between of maternal smoking in pregnancy with overweight in adulthood even adjustment for birth weight [23, 24], some publications have suggested that low birth weight has an important role in the association of fetal nicotine exposure and offspring’s overweight [25, 26].

Regarding the length/height-for-age indicator the IUGR group was different from the others at birth. At 7 and 15 days, the IUGR and tobacco were similar. However, the difference between groups disappeared after 6 months. A study conducted in the city of Pelotas, southern Brazil, found that maternal smoking during pregnancy adversely affected children’s height during early childhood, childhood, and adolescence [12]. Some mediators like Insulin, insulin-like growth factor (IGF), insulin-like growth factor binding protein-2 (IGFBP-3) show lower concentration in children of smoking mothers and, on the opposite, hemoglobin and erythropoietin have higher concentration. This could explain to fetal hypoxia produced by maternal smoking. These alterations might cause a negative effect throughout life, resulting in poor growth in childhood [13].

Conversely a cohort study conducted in England (ALSPAC) found differences in the growth patterns of children of smokers versus nonsmokers. Children of smoking mothers grew faster in early childhood, but more slowly in preschool years. However, the differences between the groups were minor and may be attributed to chance [14]. Our study found no difference in birth length between smoking and control group, both differ in the length/height-for-age only the seventh to 30 day of life. However, the difference of children in the tobacco group disappeared at 30 days of life, despite having lower birth weight compared with the control group.

In agreement with these findings another study found that the association between maternal smoking during pregnancy and overweight in male young adults could be explained in part by the parents’ socioeconomic status and by family factors that were not fully measured, such as the eating habits of the family [27].

About scholar education in our study, pregnant women of tobacco group had less education, a result similar to other studies [28]. In the United States, women with 12 or less years of education were more than 3 times as likely to smoke during pregnancy as women with more than 12 years of education [29].

The number of cigarettes smoked can influence the anthropometric measurements of infants since as some studies suggest, tobacco shows dose-response [30, 31]. In our study, pregnant women consumed an average of 10 cigarettes a day, which can be considered moderate, since most studies considered heavy smokers those with a consumption of 20 or more cigarettes/day [12, 32]. This may be one of the factors contributing to the growth trajectory of the group exposed to smoke was similar to the control.

The association between weight gain in childhood and later overweight could be partially explained by the type of diet. The use of infant formula is associated with more rapid weight gain [33]. Furthermore, breastfed infants gain weight more slowly. Breast milk production is stimulated by the suction of the baby, and therefore it would be unlikely that a rapid weight gain in breastfed infants is exclusively due to overfeeding [34]. Thus, adjusting for the type of food is important to understand the association with weight gain. The groups showed no differences in the type of food received at each follow-up time point. All groups showed reduced prevalence of exclusive breastfeeding during the follow-up period, as well as early introduction of complementary foods.

In the IUGR group head circumference at birth was significantly lower in comparison with the other groups, remaining distinct until 3rd month of life. Several studies have found that children exposed to smoking during pregnancy have a lower head circumference at birth [6, 35]. However, Matijasevich et al. [16] found that the deficits observed at birth were not present during childhood. This finding is similar to what we found, since there was no difference between the groups at 6 months.

Following up the growth of head circumference at regular time intervals makes it possible to investigate whether brain development is appropriate as there is strong correlation between head circumference growth and brain development [36]. The growth curve shows the dynamics of the global growth of the skull and its internal structures. Therefore, serial measurements facilitate early recognition of deviations in the trajectory of head growth [37] and thus, it becomes possible to carry out early intervention.

The present study has several strengths. First six measures of growth from birth to 6 months of life collected. This continuous and close approach was important because it made it possible to identify potential critical or sensitive periods for intervention, which were based on patterns of biological growth and could be predictors of overweight or obesity in childhood. Second, the method for longitudinal data analysis using GEE allowed for the analysis of continuous outcomes, even when there is missing some information regarding certain participants of the study, thus making it possible to include all participants, which may avoid bias selection.

This study also had some limitations. It was not possible to stratify the group of tobacco in relation to the gestational period of exposure. Prenatal smoking results in overall neonatal growth restriction however, in mothers who stop smoking before late pregnancy, neonates will be phenotypically similar to those who were not exposed during pregnancy, possibly decreasing the likelihood of catch-up growth [30].

Although our study did not find differences in the growth patterns of children exposed to smoking in the first months of life it has been demonstrated that the perinatal period is a key period for the development of obesity [38]. Maternal smoking during pregnancy is one of the few modifiable risk factors in the prenatal period, being the leading cause of LBW and IUGR [39, 40]. Maternal smoking may also contribute to the syndrome of sudden infant death and cause major changes in the development of the fetal nervous system [41].

Even though our findings showed no significant changes in the growth of children of smoking mothers do not rule out the possibility these effects arise at later ages. According to a study conducted in Germany, it’s was found an association of maternal smoking during the pregnancy with overweight in children. This phenomenon became evident between 4 and 6 years and, afterwards, it was accentuated until the adolescence. This and other studies have shown that the effect of maternal smoking seems to influence the development of obesity in children with increasing age [42].

In short intrauterine growth restriction seems to have more impact on the growth trajectory of the infants studied, regardless of other factors, such as smoking and diet. Whereas that, infants exposed to maternal smoking during pregnancy born with adequate weight showed the same pattern of growth control group.

## Conclusion

Considering the many adverse effects of smoking during pregnancy, women of reproductive age should be advised to stop smoking before trying to conceive, because quitting smoking after receiving the diagnosis of pregnancy may not be enough to protect their children from potential risks [43]. In addition, the harmful effects of tobacco are well known and do not apply only to somatic growth, as they affect other systems of a child’s organism.

Therefore, anti-smoking campaigns should continue to be focused on periods of increased vulnerability of the life cycle, directly or indirectly involving mothers, children, and adolescents. In a similar manner, advertising restrictions should be maintained to reduce the prevalence of consumption and, particularly, to discourage young people from having their first experience with cigarettes and other tobacco related products.

## Abbreviations

GEE: Generalized estimated equations
HIV: Human Immunodeficiency Virus
IGF: Insulin-like growth factor
IGFBP-3: Insulin-like growth factor binding protein-3
IUGR: Intrauterine growth restriction
LBW: Low birth weight
SPSS: Statistical Package for the Social Sciences
WHO: World Health Organization
